# Advanced Fabrication of 3D Micro/Nanostructures of Gallium Oxide with a Tuned Band Gap and Optical Properties

**DOI:** 10.3390/mi15030347

**Published:** 2024-02-29

**Authors:** Nishant Singh Jamwal, Amirkianoosh Kiani

**Affiliations:** 1 Silicon Hall: Micro/Nano Manufacturing Facility, Faculty of Engineering and Applied Science, Ontario Tech University, 2000 Simcoe St N, Oshawa, ON L1G 0C5, Canada; nishantsingh.jamwal@ontariotechu.net; 2 Department of Mechanical and Manufacturing Engineering (MME), Ontario Tech University, 2000 Simcoe St N, Oshawa, ON L1G 0C5, Canada

**Keywords:** gallium oxide (Ga_2_O_3_), ultra-short laser pulses, nanostructure fabrication, tuned band gap, optical properties

## Abstract

Gallium oxide (Ga_2_O_3_) is a promising material for high-power semiconductor applications due to its wide band gap and high breakdown voltage. However, the current methods for fabricating Ga_2_O_3_ nanostructures have several disadvantages, including their complex manufacturing processes and high costs. In this study, we report a novel approach for synthesizing β-Ga_2_O_3_ nanostructures on gallium phosphide (GaP) using ultra-short laser pulses for in situ nanostructure generation (ULPING). We varied the process parameters to optimize the nanostructure formation, finding that the ULPING method produces high-quality β-Ga_2_O_3_ nanostructures with a simpler and more cost-effective process when compared with existing methods. Scanning electron microscopy (SEM) and energy-dispersive X-ray spectroscopy (EDX) were used to characterize the samples, which indicated the presence of phosphorous. X-ray photoelectron spectroscopy (XPS) confirmed the formation of gallium oxide, along with a minor amount of phosphorus-containing compounds. Structural analysis using X-ray diffraction (XRD) revealed the formation of a monoclinic β-polymorph of Ga_2_O_3_. We also measured the band gap of the materials using reflection electron energy loss spectroscopy (REELS), and found that the band gap increased with higher nanostructure formation, reaching 6.2 eV for the optimized sample. Furthermore, we observed a change in the heterojunction alignment, which we attribute to the change in the oxidation of the samples. Our results demonstrate the potential of ULPING as a novel, simple, and cost-effective method for fabricating Ga_2_O_3_ nanostructures with tunable optical properties. The ULPING method offers a green alternative to existing fabrication methods, making it a promising technology for future research in the field of Ga_2_O_3_ nanostructure fabrication.

## 1. Introduction

Gallium oxide is an ultra-wide band gap semiconductor material with a reported band gap of 4.9 eV [1]. It has five different polymorphs, namely α, β, γ, δ, and ε, among which β-Ga_2_O_3_ is the most stable [2]. The other polymorphs are unstable and have poor crystalline structures; thus, less research has been conducted on them. Gallium oxide has potential in high-power applications, due to its wider band gap and very low thermal conductivity (less than half that of silicon). It has been used in several other applications, e.g., as a host material for thin-film electroluminescent displays and in field-effect transistors and gas sensors. The number of reported synthesis techniques for gallium nanostructures has increased in the last decade, since the material was first documented in the 1960s, including direct deposition, which is the most common, chemical vapor deposition, sol–gel, sputtering, and laser deposition. These techniques have been successful, and nanostructures of various sizes and shapes have been synthesized [3]. Various precursors are involved in these techniques, and they are important in the structures of the gallium oxide samples. In their review, Al-Khamis et al. mentioned a few precursors, and the various shapes and types of nanostructures generated with the employed synthesis techniques, including Ga_2_O_3_, GaN, and gallium isopropoxide. They concluded that nanowires were the most common among all the formed structures [4]. The use of amorphous gallium oxide is limited, as it is less efficient at higher powers than crystalline gallium oxide. In recent research, phase transformation from amorphous to β-Ga_2_O_3_ was performed using laser deposition. This transformation showed an increase in the material’s band gap upon increasing the substrate temperature. It was proved that, if the temperature of the laser deposition process and etching rate can be controlled, the transformation from the amorphous to polycrystalline phase can be achieved. The etching was performed in HF solution [5]. The methods for nano-synthesizing gallium oxide are multistep and require special chambers, such as with CVD and sputtering. Some laser depositions also require a solution. The processes are therefore expensive and produce harmful residuals as well.

In this work, we employed a novel synthesis technique named ULPING, which employs a solid-state pulse laser system [6]. Lasers are common in applications such as cutting, laser printing, heat treatment, communication, etc. [7,8,9]. Lasers can be categorized based on their operational mode as continuous-wave (CW) or pulsed lasers. CW lasers deliver continuous energy onto the surface of the material, whereas pulsed lasers emit energy in pulses [10,11,12]. Herein, we used picosecond laser pulses for the nanostructure’s generation. The major focus was on short high-energy pulses, which produce enough energy to ablate the sample, providing a short gap for the nanostructures to form over the surface. There are several advantages of using laser pulses, including the ability to control the energy of the pulse. The energy can be made precise based on the desired conditions, which, in turn, reduces any unnecessary heating of the material. This prevents the material from degrading. The substrate remains undamaged, allowing for the creation of nanostructures with fewer defects, a higher surface area, and an improved crystal structure [13,14,15,16]. Overall, this work provides a new and promising approach for synthesizing gallium oxide nanostructures, which may have potential applications in high-power devices and other fields.

## 2. Methodology

**Figure 1:**
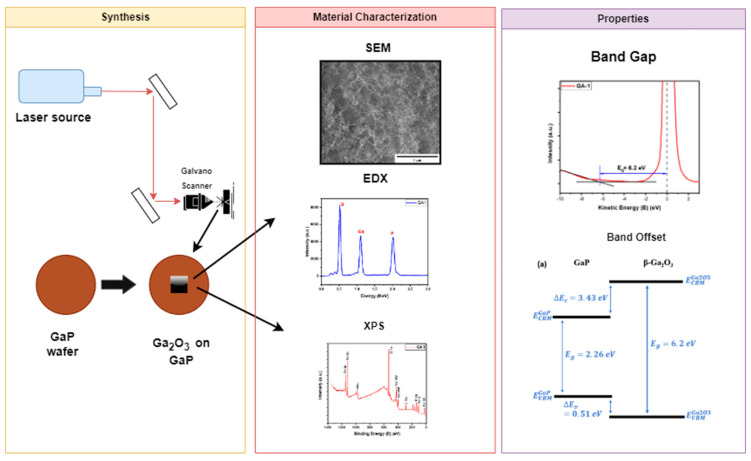
The steps in the synthesis, characterization, and testing of the gallium-oxide-nanostructured samples. Each parameter was varied in three sets of samples, and a common set of parameters (Ga1) was used for all the sets (Table 1). We characterized the synthesized nanoparticles using several analytical techniques, including scanning electron microscopy (SEM), energy-dispersive X-ray spectroscopy (EDX), X-ray photoelectron spectroscopy (XPS), reflection electron energy loss spectroscopy (REELS), and X-ray diffraction (XRD). The samples were sputtered with platinum before SEM and EDX analysis.

In this study, we employed the ultrashort pulsed laser-induced nanoparticle generation (ULPING) technique for the synthesis of nanoparticles on a gallium phosphate (GaP) substrate. A pulsed fiber laser (IPG Model: YLPP-1-150V-30) was used to perform the ULPING technique. The shape and design of the synthesis were defined using Marking Mate software (Version 2.7), which allowed us to preview the outlining area (see Figure 1). The sample was positioned 2.3 cm from the Galvano scanner. We synthesized nine samples by varying four laser parameters as follows: power, pulse repetition, pulse duration, and the scan speed of the Galvano scanner. The whole process was performed in ambient room conditions without a vacuum chamber.

Table 1 summarizes the sample types, and the ULPING parameters varied in each set. The best sample was chosen for XRD analysis, which was performed using a Rigaku (Tokyo, Japan) Miniflex 600 diffractometer with a Cu Kα radiation source (λ= 1.5406 Å).

Herein, the SEM and EDX were performed using Thermofisher Quanta 3D LSB equipment. XPS and REELS were carried out with the help of Thermo Scientific Nexsa G2 equipment.

## 3. Statistical Analysis

Data processing and analysis were performed using Origin Pro 2021a software (OriginPro: 9.8), which was also used for graph plotting. Data smoothing was performed using the weighted average method in Origin Pro 2021a for noise reduction. Peak fitting for the XPS data was carried out using Avantage software (2022), and the calibration of XPS spectra was also carried out with Avantage, using the adventitious carbon located at 284.8 eV. The Shirley background type was used for XPS graphs. ImageJ was used to add scale bars to the SEM images. The results presented herein are reproducible. The data were obtained after preparing three sets of samples and performing multiple measurements. The error margin for the processed data was less than 5%.

## 4. Results and Discussion

### 4.1. Material Characterization Using SEM, EDX, and XPS

We analyzed the changes in the morphology and properties of the materials after varying four different laser parameters. This comparison was conducted on a set-by-set basis. We compared the properties of GA1, the best-performing sample, with the properties of the other samples in the same set.

### 4.2. Power-Varied Samples

**Figure 2:**
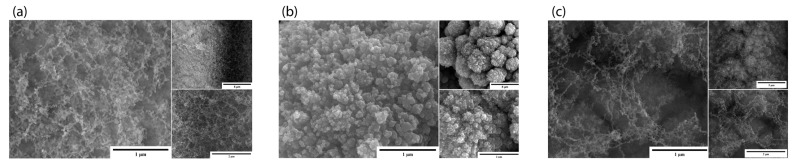
SEM images of power-varied samples. (**a**) GA1: 1 µm (50,000×), 5 µm (10,000×), and 2 µm (35,000×); (**b**) GA2: 1 µm (50,000×), 5 µm (10,000×), and 2 µm (35,000×); (**c**) GA3: 1 µm (50,000×), 5 µm (10,000×), and 2 µm (35,000×).

In the samples where power was varied, we observed that GA1 displayed the best nanostructures (Figure 2a). The formed nanostructures were 3D nano-network structures like those previously reported by our group for other materials [9]. In this set of samples, we observed that at a power of 2 W, the ablation was insufficient to form enough nanostructures; the SEM images demonstrate that GA3 (Figure 2c) had fewer nanostructures than GA1. GA2, where we used the highest power, showed an agglomeration of the nanostructures. Due to the higher power, the nanostructure density increased, and they formed an agglomerate.

**Figure 3:**
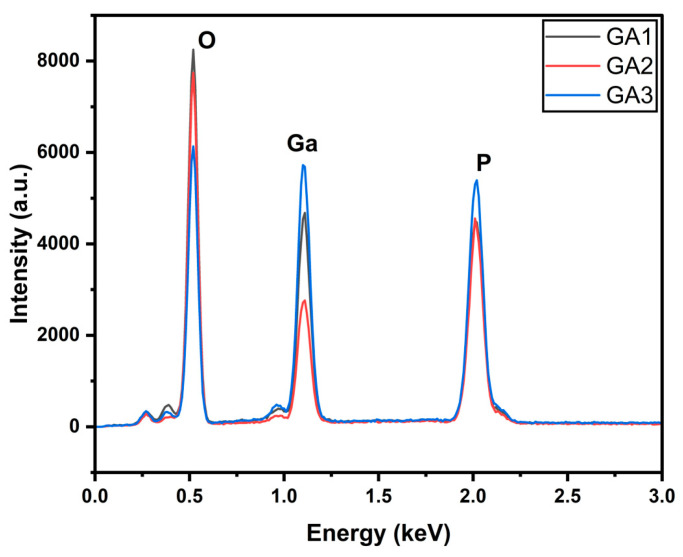
EDX of power-varied samples.

The EDX in Figure 3 illustrates the presence of gallium, oxygen, and phosphorus. It indicates that the surface was oxidized. The intensity of the oxygen peak is higher for GA1 than for the other two.

**Figure 4:**
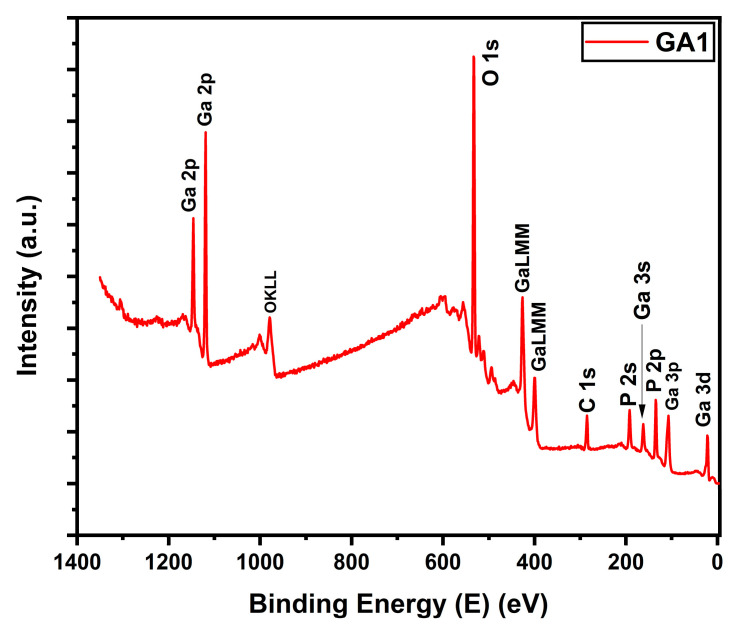
XPS survey for GA1.

**Figure 5:**
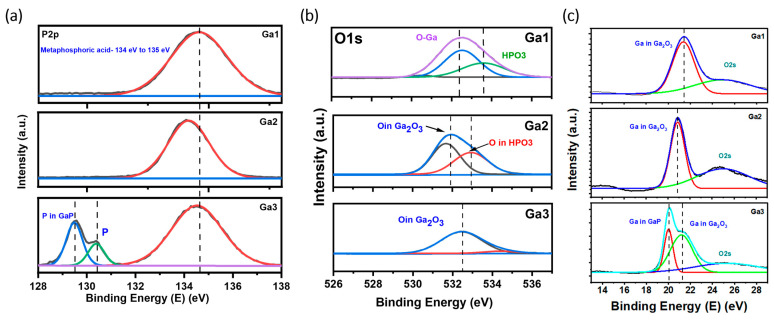
Deconvoluted XPS peaks for power-varied samples. (**a**) P2p peaks. (**b**) O1s peaks. (**c**) Ga3d peaks.

X-ray photoelectron spectroscopy (XPS) was utilized to identify the formed compound, and determine the corresponding ratio that best explained the formation of gallium oxide. The XPS survey (Figure 4) showed similar peaks for Ga, O, and P, along with carbon, which was employed to authenticate the collected XPS data. By deconvoluting the XPS peaks for each element, the satellite peaks and formed compounds were identified based on the binding energies of the peaks. Figure 5a presents the P core-level spectra for the power-varied samples. In GA1, where the most nanostructures were observed in the SEM images, the formation of metaphosphoric acid was noted. The peaks for GA1, GA2, and GA3 were observed at 134.6 eV, 134.1 eV, and 134.5 eV, respectively, which corresponds to metaphosphoric acid. In its pure form, metaphosphoric acid is typically a glassy (solid) substance at room temperature. In its anhydrous state, or when freshly prepared, it is solid under standard room conditions.

The oxidation of GaP by oxygen in ambient air can result in the formation of gallium oxide. Phosphorous species were also present, which, upon deconvoluting the P2p spectra, indicated metaphosphoric acid. We employed a pulsed laser, which can produce high-energy conditions, and, in turn, promote the formation of reactive species. This simple reaction results in the formation of Ga_2_O_3_ and P_4_O_6_ upon an increased temperature in an ambient atmosphere, as shown in Equation (1). This applies to the samples with 5 W and 8 W powers. However, P and GaP core-level spectra peaks were observed in GA3, indicating that the laser material interaction was insufficient for the complete formation of gallium oxide.

(1) $$4GaP+15\;O_{2}--\to 2{Ga}_{2}O_{3}+4P_{4}O_{6}\;$$

This was confirmed by the O1s and Ga3d peaks, which showed gallium bonding with oxygen, indicating the formation of a metal oxide, along with a satellite peak for metaphosphate, as shown in Figure 5b. In the Ga3d graph, gallium oxide was observed for GA1 and GA2, whereas, for GA3, a small peak for GaP was also observed. Observing the oxygen peak at approximately 532 eV [17], it can be inferred that the oxygen atom is coordinated with Ga in a tetrahedral configuration, which is evident in the β-polymorph of gallium oxide. A chemical shift from 20 eV to approximately 22 eV was observed for GaP in the Ga3d peaks, due to the formation of complex bonds between the oxygen atoms and the core–shell electrons of gallium. This is because of the electronegativity of oxygen atoms, which shifts to a higher energy, thus resulting in the trivalent state of Ga [18].

### 4.3. Scan Speed Samples

The samples were prepared with three scanning speeds. The scanning speed is the speed of the Galvano scanner, which defines how fast the pattern added onto the substrate is finished.

**Figure 6:**
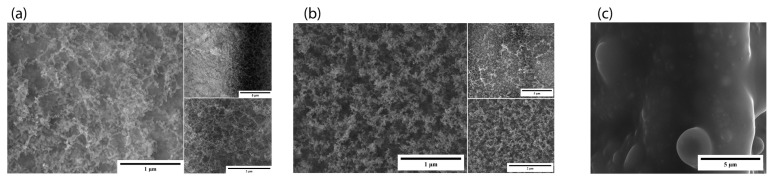
SEM images of scanning-speed-varied samples. (**a**) GA1: 1 µm (50,000×), 5 µm (10,000×), and 2 µm (35,000×); (**b**) GA4: 1 µm (50,000×), 5 µm (10,000×), and 2 µm (35,000×); (**c**) GA5: 1 µm (50,000×), 5 µm (10,000×), and 2 µm (35,000×).

Nanostructures are visible in the SEM images of the GA4 sample, where the slowest scanning speed was applied; however, there are more clusters of nanostructures at 50,000× magnification (Figure 6b). Meanwhile, we observed no nanostructure formation in GA5 (Figure 6c), where the scanning speed was highest, and the irradiation was unsuccessful. Better nanostructures were observed in GA1, where the scanning speed was in between. The laser material contact depends on how fast the Galvano scanner moves to scan the material. The scanning speed was high at 50 mm/s; therefore, we could not achieve the desired energy on the sample. There was irradiation; however, there was insufficient energy to disintegrate the surface and form nanostructures. The scanning speed was slow in GA4, therefore, at one point, more energy was concentrated on the sample. This resulted in more irradiation, and, thus, cluster formations occurred.

**Figure 7:**
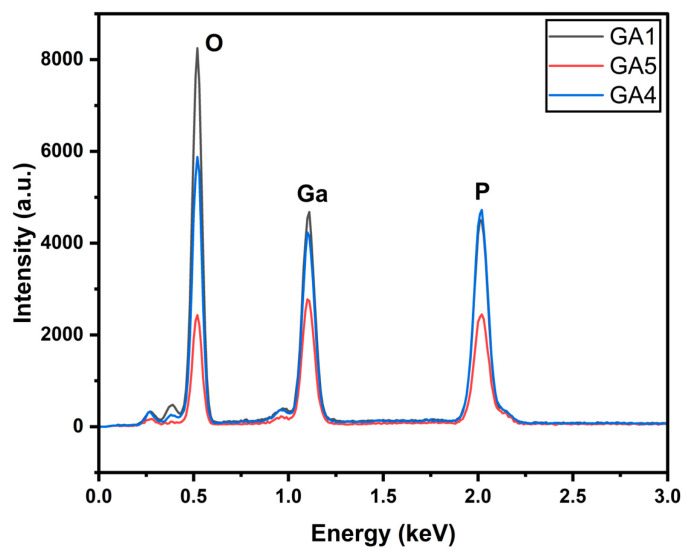
EDX of scanning-speed-varied samples.

The EDX graph in Figure 7 shows higher oxidation rates in GA1 and GA4 compared with GA5, which did not exhibit any nanostructures. Although GA5 exhibited lower oxidation, it also showed lower Ga and P peaks, which can lead to the formation of gallium oxide.

**Figure 8:**
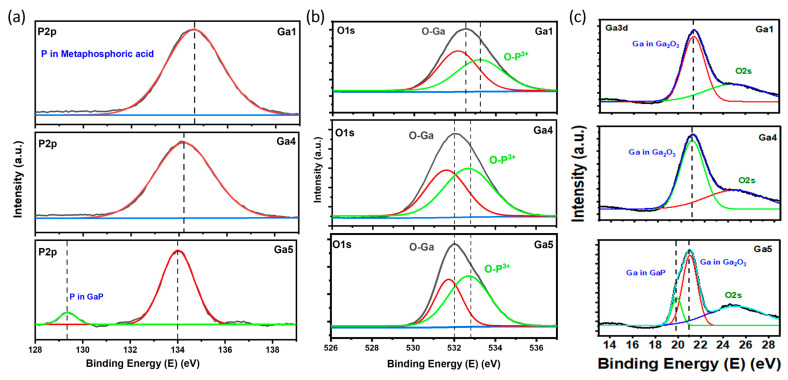
Deconvoluted XPS peaks for scanning-speed-varied samples. (**a**) P2p peaks. (**b**) O1s peaks. (**c**) Ga3d peaks.

In the EDX and XPS of the samples in Figure 7 and Figure 8, oxidation is visible for all of the samples, and peaks are visible for Ga, O, and P. The deconvoluted XPS peaks show a similar trend to the previous set (the power-varied samples). The peaks at a higher scanning speed (GA5) indicate the presence of GaP. Unlike GA3, where we used a lower average power and obtained nanostructures, we did not obtain nanostructures in this case. However, we did achieve the oxidation and formation of gallium oxide, due to the pulse energy, which was constant for GA1, GA4, and GA5.

### 4.4. Pulse-Repetition-Varied Samples

**Figure 9:**
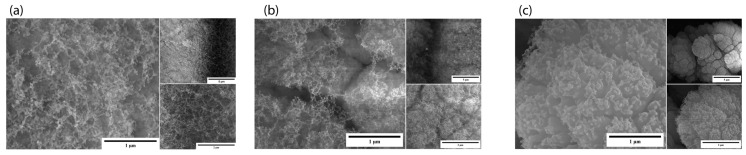
SEM images of power-varied samples. (**a**) GA1: 1 µm (50,000×), 5 µm (10,000×), and 2 µm (35,000×); (**b**) GA6: 1 µm (50,000×), 5 µm (10,000×), and 2 µm (35,000×); (**c**) GA7: 1 µm (50,000×), 5 µm (10,000×), and 2 µm (35,000×).

We observed better fibers in GA1 at 1200 kHz than in GA6 and GA7, where we varied the pulse repetition rate, as shown in Table 1. We observed that the growth of nanostructures was greatly affected by the pulse repetition rate, such as in GA7 (Figure 9c), where we did not observe nanostructures, but micron-size structures were visible. This is because of the energy delivered to the material. Due to the lower rate, the irradiation rate was inefficient for the formation of nanostructures. Some nanostructures were generated in GA6 as we increased the repetition rate, which may also be due to the different heating and cooling rates of the material. The rate of heating and cooling cycles is rapid at a higher pulse repetition, so the structural formation varies.

**Figure 10:**
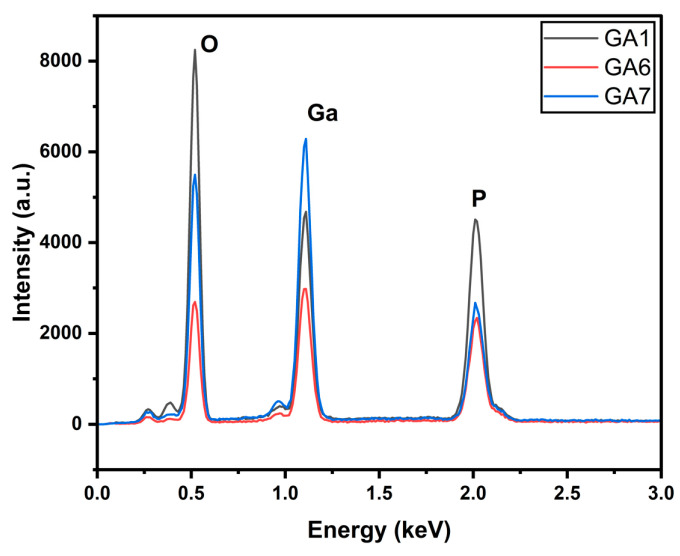
EDX of pulse-repetition-varied samples.

**Figure 11:**
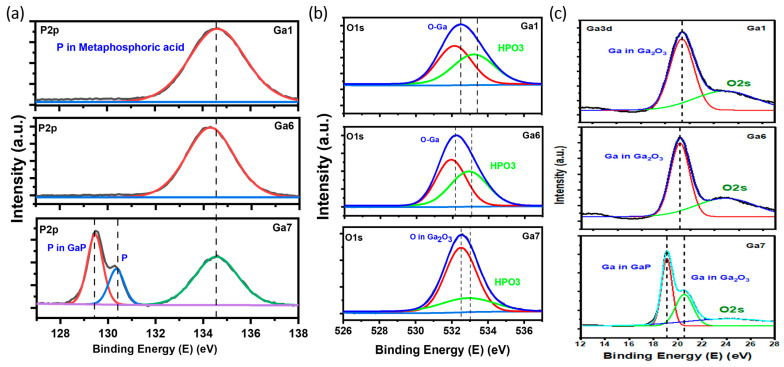
Deconvoluted XPS peaks for pulse-repetition-varied samples. (**a**) P2p peaks. (**b**) O1s peaks. (**c**) Ga3d peaks.

The EDX in Figure 10 and the deconvoluted XPS peaks in Figure 11 show the presence of three elements. The XPS peaks show a very high peak for GaP in Ga3d in the GA7 sample. This is due to the lower pulse repetitions, meaning the irradiation was worse than in GA6 and GA1. Similar to the power-varied samples, in GA7, we obtained elemental phosphorous, along with metaphosphoric acid. This was also due to inefficient irradiation.

### 4.5. Pulse-Duration-Varied Samples

**Figure 12:**
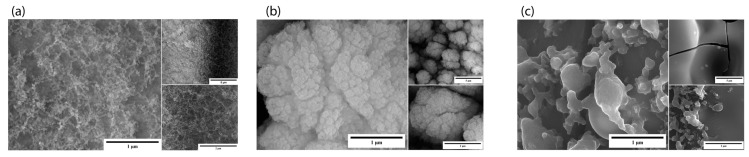
SEM images of power-varied samples. (**a**) GA1: 1 µm (50,000×), 5 µm (10,000×), and 2 µm (35,000×); (**b**) GA8: 1 µm (50,000×), 5 µm (10,000×), and 2 µm (35,000×); (**c**) GA9: 1 µm (50,000×), 5 µm (10,000×), and 2 µm (35,000×).

GA1, GA8, and GA9 were compared to observe the variation in the pulse duration. We used a shorter pulse duration for GA1, and higher durations for GA8 and GA9. The SEM images in Figure 12 show that we obtained nanostructures with a shorter pulse duration. As we increased the duration, the laser–material interaction was not precise for nano-synthesis. This is because the energy transfer is more efficient with shorter pulse durations, due to multiphoton absorption and tunneling ionization. We can observe a crack line in GA9, which is due to thermal damage from the longer pulse duration [19]. Shorter pulse durations generate shorter bursts of energy, which then confines the generated heat to a smaller region. That is why we obtained a better structure in GA1.

**Figure 13:**
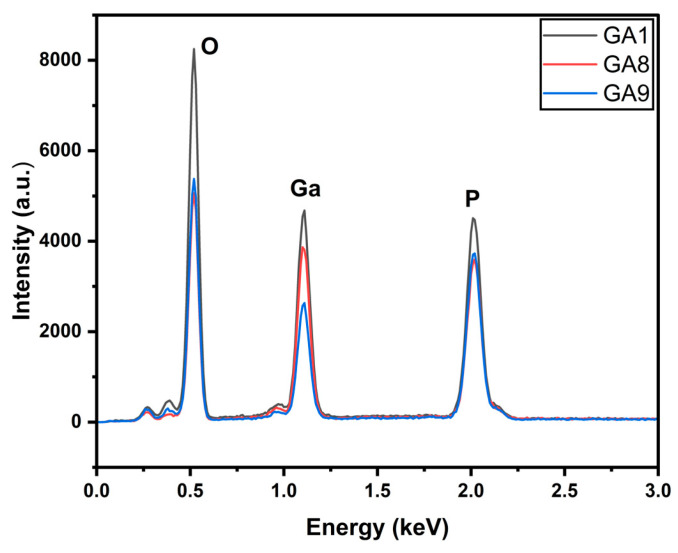
EDX of pulse-duration-varied samples.

**Figure 14:**
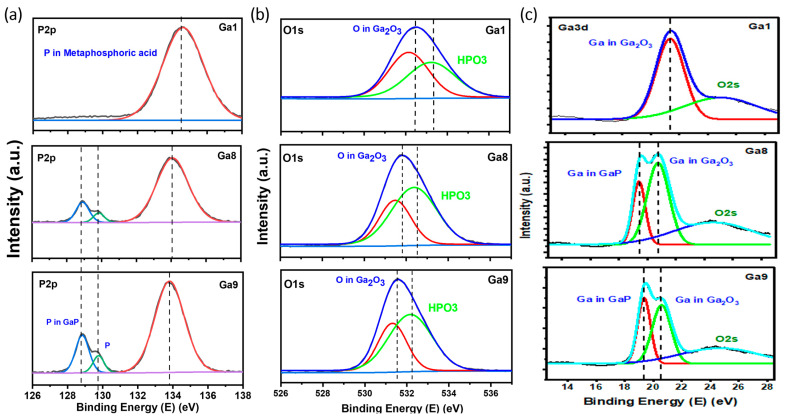
Deconvoluted XPS peaks for pulse-duration-varied samples. (**a**) P2p peaks. (**b**) O1s peaks. (**c**) Ga3d peaks.

Similar to the previous sets, the EDX in Figure 13 shows the presence of three elements, O, Ga, and P. A similar trend is observed, where the samples with more nanostructures have higher oxidation peaks. The XPS peaks in Figure 14 illustrate the formation of gallium oxide in all the samples. We observed Ga core spectra peaks corresponding to GaP in GA8 and GA9. The peak for GaP had a higher intensity than that for GA1 with lower irradiation. The reaction and, thus, the formation of gallium oxide, remained incomplete.

### 4.6. XRD of GA-1

**Figure 15:**
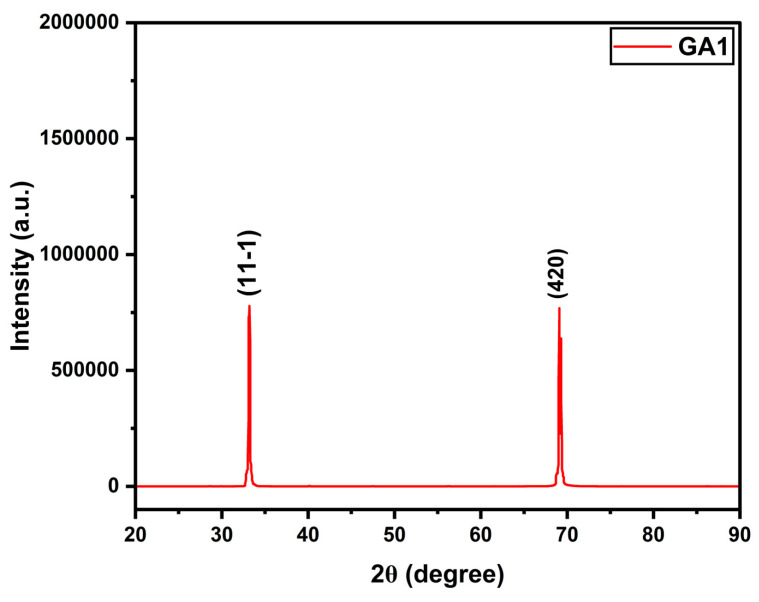
XRD for GA1 sample.

Our preliminary analysis of the XPS data suggests evidence of crystalline structure formation in the gallium oxide nanostructure and polymorph. Further investigation is needed to comprehensively study and discuss these findings in future studies. XRD was carried out on the best sample, GA1. As seen in Figure 15, we determined peaks at approximately 33.2° and 69.06°, corresponding to crystalline β-Ga_2_O_3_. The positions and intensities of the peaks contributed to the crystal lattice and orientation of the planes in the formed nanostructures of gallium oxide. From (11-1), it can be inferred that the nanostructures had a monoclinic crystal structure, and (420) corresponds to a hexagonal crystal structure. The degrees suggest that the nanostructures were small, as the peaks were broadened due to the size limitation of the crystallites. The peaks agree with the JCPDS card no. 41-1103 for a monoclinic lattice [20].

Bragg’s law for diffraction is expressed in Equation (2),

(2) $$d_{\left(hkl\right)}=n\lambda /2sin\theta$$

where $d_{\left(hkl\right)}$ is the d-spacing of the lattices (11-1 and 420, in this case), n is the order of diffraction (n = 1), and λ is the X-ray wavelength, which was 1.5406 Å. Using the equation, we achieved $d_{\left(11-1\right)}$ = 2.696 Å, which is slightly larger than the d_(JCPDS)_ value of 2.676 [21]. This indicates the nanostructures had a higher quality.

Scherrer’s Equation (3) was employed as follows:

(3) $$C_{s}=0.9\lambda /\beta \;cos\theta$$

where C_s_ is the crystalline size in nm, and β is the full width at half maximum (FWHM). The crystalline size of GA1 was small, at 8.87 nm. A small crystalline size can help create more reactive sites, which can be advantageous for sensor applications.

### 4.7. Band Gap

We observed the formation of gallium oxide in all the samples; however, the formation of nanostructures is an important parameter in determining the properties of a material. The band gap is the most promising property of a semiconductor. REELS was employed to determine the material’s band gap (Table 2).

**Figure 16:**
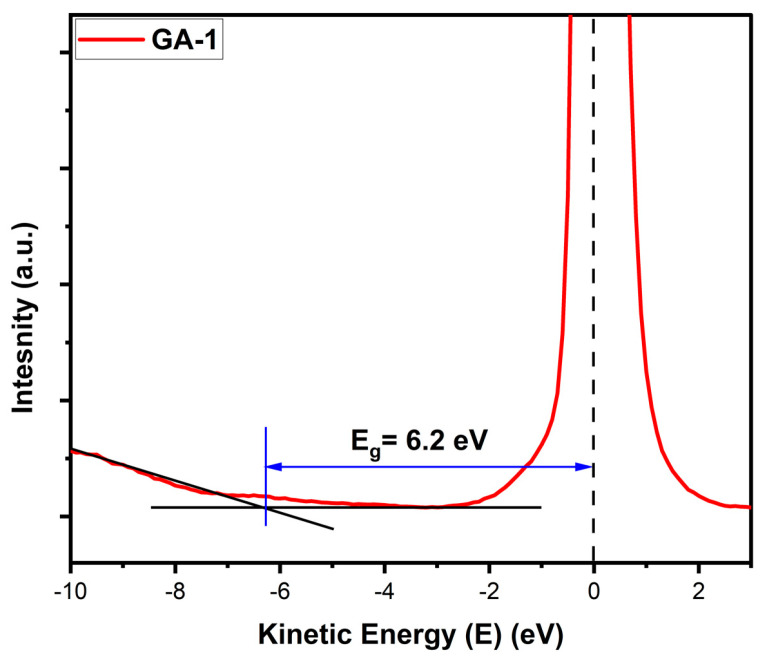
Band gap determination using REELS data for GA1.

The REELS data were plotted, interpolation was performed, and the band gap was determined, as illustrated in Figure 16.

All the band gap values were in the range of 3.78 to 6.2 eV, which shows that the band gap varied with the varying structures. The band gap for GA1 was the highest among all the samples, and it was higher than the general band gap value of gallium oxide (4.8 eV). This is due to the nanostructures formed. The formation of nanostructures increases the band structure and forms discrete bands, which increases the band gap of the material [22]. The band gap range agreed with that of gallium oxide. The increased band gap in nanostructures results from quantum confinement.

### 4.8. Band Offset

Band offset is an important parameter for a heterojunction formed from semiconductors. The band offset can greatly affect the properties of the material. The data collected from XPS were used to determine the band offset.

**Figure 17:**
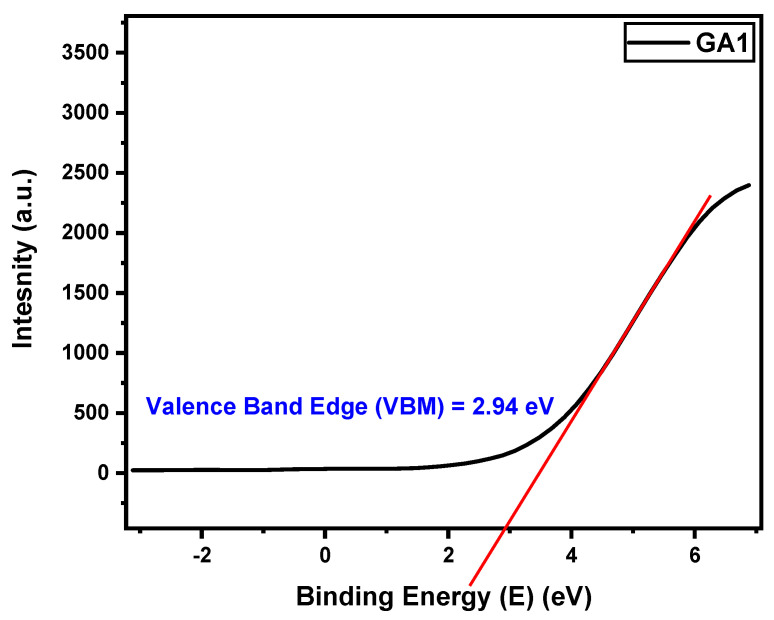
Valence band edge determination for GA1 sample from the valence band XPS spectra.

Initially, the valence band spectra were used to determine the valence band maximum (VBM) for the samples. This was performed as shown in Figure 17. The values are tabulated in Table 2.

The band gaps of the samples were determined, and the XPS provided the core-level binding energies. Using Kraut’s method [23], the valence band offset (VBO) and the conduction band offset (CBO) can be derived with

(4) $$∆E_{v}=\left(E_{Ga\;2p}^{Ga2O3}-E_{VBM}^{Ga2O3}\right)-(E_{Ga\;2p}^{GaP}-E_{VBM}^{GaP})-(E_{Ga\;2p}^{Ga2O3}-E_{Ga\;2p}^{GaP})$$

where $E_{Ga\;2p}^{Ga2O3}$ and $E_{Ga\;2p}^{GaP}$ are the binding energy of gallium oxide and GaP 2p_3/2_ peaks. $E_{VBM}^{Ga2O3}$ and $E_{VBM}^{GaP}$ are the binding energies for the VBM of Ga_2_O_3_ and GaP, measured using the valence band spectra from XPS. The band gap of GaP was 2.26 eV, and the VBM was 3.45 eV. The Ga2p core-level binding energy for GaP was 1116.5 eV.

**Figure 18:**
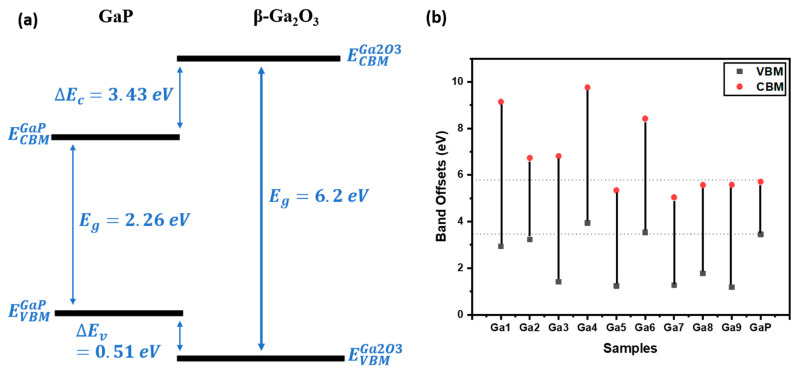
Band offset with respect to GaP. (**a**) Band offset diagram for GA1, which depicts the type-I heterojunction alignment. (**b**) Band offset for all the samples depicting the variation in VBM and CBM.

These values were substituted into Equation (4), and the VBO was determined. Similar to finding the conduction band offset (CBO) [24], Equation (4) can be used as follows:

(5) $${∆E}_{c}=E_{g}^{Ga2O3}-E_{g}^{GaP}-∆E_{v}$$

where $E_{g}^{Ga2O3}$ and $E_{g}^{GaP}$ are the band gaps of gallium oxide and GaP. Three different types of heterojunction interfaces were noted upon tabulating the data (Table 3) and preparing the band diagram in Figure 18. In general, gallium oxide and GaP have a type-III heterojunction alignment as the CBM of gallium oxide is higher than that of GaP. However, the VBM of GaP is higher than that of gallium oxide. As indicated in the table, the samples with a lower oxidation level had a type-II alignment. The samples with higher oxidation levels had a type-I alignment (as shown for GA1 in Figure 18a). The other samples had a type-III alignment. This is depicted in Figure 18b, demonstrating that the conduction band offset increased as the oxidation increased. A shift to a type-I alignment was exhibited in the power samples. The formation of heterojunctions is a complex process, and includes factors such as the orientations of the crystal, the surface preparation, and the presence of defects and impurities [25,26]. Thus, it is hypothesized that the change is due to oxidation [27,28]. Having a thicker native layer affects the material’s VBO and CBO, leading to a change in the alignment.

## 5. Conclusions

This work concludes that ULPING is effective for synthesizing gallium oxide nanostructures on a gallium phosphide (GaP) substrate. In this study, we were able to synthesize gallium oxide on GaP in all the samples. We observed nanostructures in GA1, GA3, GA4, and GA6 by varying the laser parameters. GA1 had the best nanostructures. The nanostructures occurred in a uniform layer. Thus, the parameters used in GA1 are the best to synthesize gallium oxide nanostructures. Material characterization techniques including XPS and XRD confirmed that the formed structure was β-Ga_2_O_3_, which is crystalline. When deconvoluting the XPS peaks, we also observed the formation of metaphosphoric acid in a very small quantity. This metaphosphoric acid can be removed using several techniques, such as thermal annealing, acid cleansing, and anodization. We recommend thermal annealing as other techniques can affect the formed nanostructures. However, thermal annealing at a higher temperature can affect the gallium oxide nanostructure. Using a low annealing temperature below 500 °C can help preserve the nanostructure and remove the metaphosphoric acid. The nanostructured samples showed a wider band gap than the samples without nanostructures. In general, the band gaps were between 3.78 and 6.8 eV. Defining the band offset is an important feature, with which the alignment of the heterojunction between the two materials can be defined. All three alignment types were observed herein, and a hypothesis was made based on the oxidation of the samples. A higher oxidation resulted in the formation of type-I heterojunctions. However, this can be researched further, alongside the other conditions, for the change in the alignment.

## Figures and Tables

**Table 1:** Sample types and varied ULPING parameters.

	Sample	Power (W)	Pulse Repetition (kHz)	Scan Speed (mm/s)	Pulse Duration (Picosecond)
	GA1	5	1200	20	150
Varied power	GA2	8	1200	20	150
	GA3	2.5	1200	20	150
Varied scan speed	GA4	5	1200	10	150
	GA5	5	1200	50	150
Varied pulse repetition	GA6	5	900	20	150
	GA7	5	600	20	150
Varied pulse duration	GA8	5	1200	20	1000
	GA9	5	1200	20	5000

**Table 2:** Band gaps of all the samples measured using REELS.

Sample	Band Gap (eV)
Ga1	6.2
Ga2	3.5
Ga3	5.4
Ga4	5.82
Ga5	4.1
Ga6	4.88
Ga7	3.78
Ga8	3.8
Ga9	4.4

**Table 3:** Data on band offset and heterojunction alignment types.

Sample	Band Gap of Ga_2_O_3_ (eV)	VBM of Ga_2_O_3_ (eV)	CBM of Ga_2_O_3_ (eV)	Ga2p of Ga_2_O_3_ (eV)	VBO	CBO	Type of Heterojunction Alignment
GA1	6.2	2.94	9.14	1119.53	0.51	3.43	I
GA2	3.5	3.23	6.73	1118.76	0.22	1.02	I
GA3	5.4	1.41	6.81	1118.31	2.04	1.1	I
GA4	5.82	3.94	9.76	1119.02	−0.49	4.05	III
GA5	4.1	1.24	5.34	1118.87	2.21	−0.37	II
GA6	4.88	3.54	8.42	1119.22	−0.09	2.71	III
GA7	3.78	1.26	5.04	1118.18	2.19	−0.67	II
GA8	3.8	1.77	5.57	1118.73	1.68	−0.14	II
GA9	4.4	1.18	5.58	1118.48	2.27	−0.13	II

## Data Availability

The data can be provided upon request.

## Abbreviations

ULPING	Ultra-short laser pulses for in situ nanostructure generation
SEM	Scanning electron microscopy
EDX	Energy-dispersive X-ray spectroscopy
XPS	X-ray photoelectron spectroscopy
XRD	X-ray diffraction
REELS	Reflection electron energy loss spectroscopy
GaP	Gallium phosphide
VBM	Valence band maximum
CBM	Conduction band minimum
VBO	Valence band offset
CBO	Conduction band offset
